# Optimal regimens based on PK/PD cutoff evaluation of ceftiofur *against Actinobacillus pleuropneumoniae* in swine

**DOI:** 10.1186/s12917-020-02589-9

**Published:** 2020-09-29

**Authors:** Da Sun, Kun Mi, Haihong Hao, Shuyu Xie, Dongmei Chen, Lingli Huang

**Affiliations:** 1 National Reference Laboratory of Veterinary Drug Residues and MAO Key Laboratory for Detection of Veterinary Drug Residues, Huazhong Agricultural University, Wuhan, China; 2 Department of Veterinary Pharmacology, College of Veterinary Medicine, Huazhong Agricultural University, Wuhan, China

**Keywords:** Ceftiofur, *Actinobacillus pleuropneumoniae*, Epidemiologic cutoff value, Pharmacokinetic/pharmacodynamics cutoff, Dosage

## Abstract

**Background:**

*Actinobacillus pleuropneumoniae* formerly known as *Haemophilus pleuropneumoniae*, can cause pleuropneumoniae in pigs, which lead to significant mortality. Ceftiofur was the first cephalosporin antibiotic used in animals, which was effective against gram-negative and gram-positive bacterium. This study aimed to formulate a rational dosage strategy and review the preceding recommended dosage based on PK/PD modeling and Establish Clinical breakpoint of ceftiofur against *Actinobacillus pleuropneumoniae* based on the pharmacodynamic-pharmacokinetic cutoff.

**Results:**

The epidemiologic cutoff value was 0.125 μg/mL. The results of the pharmacodynamic study showed that the MICs of BW39 were 0.5 μg/mL and 1 μg/mL in vitro and ex-vivo, respectively. The minimal bactericidal concentrations (MBCs) under in vitro and ex vivo conditions were both 1 μg/mL. The time-killing profiles of ceftiofur against BW39 were time-dependent with a partly concentration-dependent pattern. Based on the inhibitory sigmoid E_max_ model, the AUC_24 h_/MIC values for the bacteriostatic, bactericidal, and elimination effects in serum were 45.73, 63.83, and 69.04 h for healthy pigs separately. According to the Monte Carlo simulation, the CO_PD_ was calculated as 2 μg/mL, and the optimized dosage regimen of ceftiofur against *Actinobacillus pleuropneumoniae* to achieve bacteriostatic, bactericidal, and elimination effects over 24 h was 2.13, 2.97, and 3.42 mg/kg for the 50% target attainment rate (TAR) and 2.47, 3.21, and 3.70 mg/kg for the 90% TAR respectively.

**Conclusions:**

In conclusion, we reveal the EOFF and PK/PD cutoff values of ceftiofur against A. pleuropneumoniae in piglets. However, with the paucity of clinical data for ceftiofur to establish a clinical cutoff against A. pleuropneumoniae, the PK/PD cutoff value of 2 μg/mL will be recommended as surrogate. According to the PK/PD data and the MIC distribution in China, the single bactericidal dose was 3.21 mg/kg for the 90% target, which would be more able to cure *Actinobacillus pleuropneumoniae* and avoid the emergence of resistance for clinical ceftiofur use in piglet.

## Background

Porcine pleuropneumonia caused by *Actinobacillus pleuropneumoniae* is a highly contagious respiratory disease that causes hemorrhage, purulent and fibrous pleuropneumonia. The disease is widely distributed and bring severe losses to the pig industry. The morbidity of the disease can be as high as 100%, but usually varies from 30 to 50% [1]. There are currently 18 serotypes of *Actinobacillus pleuropneumoniae* [2, 3], and the prevalent serotypes vary in different countries and regions, which may lead to piglet infected with multiple serotypes.

Ceftiofur was the first third-generation cephalosporin antibiotic used in animals. It is effective for gram-negative bacteria and gram-positive bacteria and has a good clinical therapeutic effect against animal respiratory diseases [4]. The FDA has approved ceftiofur hydrochloride, ceftiofur sodium and ceftiofur crystal-free acid for the treatment of respiratory diseases of pigs, cattle, horses, goats and sheep [5–7].

Clinical breakpoint are MIC values, which are used in clinical microbiology laboratories to categorize microorganisms as clinically [8]. Establishing clinical breakpoint contains three parts: (i) an epidemiological cut-off value (ECOFF), described by EUCAST as the ECOFF, it defines the upper end(> 95%) of the wild type MIC distribution. (ii) PK/PD breakpoint set by EUCAST is generally taken as highest MIC for which a selected PK/PD index can be achieve in the target population, given the standard regimes and taking into account the lower 95–99% prediction intervals for the population. (iii) clinical cut-off described by EUCAST in human medicine, it is related to clinical outcomes(cure vs non-cure) and requires specific investigations during prospective clinical trials [9]. Although the CLSI has established the breakpoint of ceftiofur against *Actinobacillus pleuropneumoniae*, considering that the use of antibiotics and drug resistance in China is totally different, it is necessary to formulate the relevant criteria for ceftiofur against *Actinobacillus pleuropneumoniae* in China based on the local situation.

The abuse of antibiotics and unreasonable doses of antimicrobial agents are the main factors, which contribute to the development of resistance [10, 11]. Existing data have suggested that the resistance rate of *Actinobacillus pleuropneumoniae* to tetracyclines and penicillin reached 90% and showed different degrees of resistance to macrolides and aminoglycosides. However, *Actinobacillus pleuropneumoniae* is still highly sensitive to ceftiofur [12–14] and Clinical breakpoint should be set up for ceftiofur against *Actinobacillus pleuropneumoniae*. Therefore, we need to use scientific methods to determine the reasonable dosage of ceftiofur and establish drug resistance criteria to protect the clinical efficacy of ceftiofur. Previous PK study [15] has shown ceftiofur sodium and ceftiofur hydrochloride has similar therapeutic efficacy. And the recommended dosage of ceftiofur for the treatment of swine respiratory disease in the U.S. and in Europe ranged from 3 to 5 mg of ceftiofur equivalents (CE)/kg body weight. PK/PD integration modeling data can provide an optimal drug dosage strategy, reducing resistance development, which is a key method to evaluate the clinically relevant relationship between time, drug concentration, and effect. However, there are no data supporting the rational dosage of ceftiofur against *Actinobacillus pleuropneumoniae* based on the PK/PD model in China.

The purposes of this investigation were to (i) study the pharmacokinetic/pharmacodynamic of ceftiofur against *Actinobacillus pleuropneumoniae*, (ii) formulate a rational dosage strategy and review the preceding recommended dosage based on PK/PD modeling for ceftiofur against *Actinobacillus pleuropneumoniae*. (iii) establish a clinical breakpoint based on pharmacokinetic/pharmacodynamic cutoff values.

## Results

### MIC distribution and epidemiologic cutoff value

**Fig. 1:**
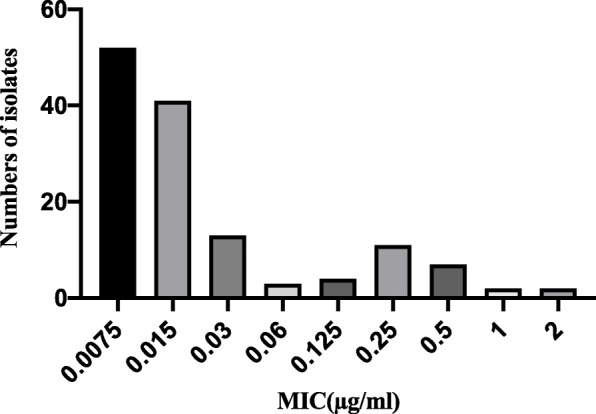
Distribution of MIC for ceftiofur against 135 strains *Actinobacillus pleuropneumonia*

The MIC distribution of ceftiofur against *Actinobacillus pleuropneumoniae* is shown in Fig. 1. The MIC values ranged from 0.0075 to 4 μg/mL. The MIC_50_ and MIC_90_ were calculated to be 0.015 and 0.5 μg/mL. We selected the serotype 1 strain *App BW39* whose MIC was similar to MIC90(0.5 μg/mL) to analyzed for PD study in broth and serum. The ECV was 0.125 μg/mL, encompassing 99.9% of the wild-type isolates.

### MICs, MBCs, MPC and PAE

The MIC values of ceftiofur against *App* BW39 were 0.5 μg/mL and 1 μg/mL in vitro and ex vivo, and the MBC values were both 2 μg/mL in vitro and ex vivo*.* The MBC/MIC ratios were 2 and 1 in vitro and ex vivo indicating that ceftiofur is a bactericidal drug. The MPC value of ceftiofur against *App* BW39 was 1.6 μg/mL, and the MSW was 1–1.6 μg/mL. The post antibiotic effect (PAE) values after exposure to the concentrations of 1MIC, 2MIC and 4MIC were 0.33, 0.41 and 0.66 h for 1 h exposure and 0.73, 0.92 and 1.17 h for 2 h exposure, respectively (Table 1). The result suggested PAE had a positive correlation with exposure time. PAE was also increased with drug concentration, but the degree of enhancement was weak.

**Table 1:** Post antibiotic effect (PAE) after 1 h and 2 h

Ceftiofur	1 h	2 h
(μg/mL)	(h)	(h)
MIC	0.33	0.73
2MIC	0.41	0.92
4MIC	0.66	1.17

### Time-killing curves

**Fig. 2:**
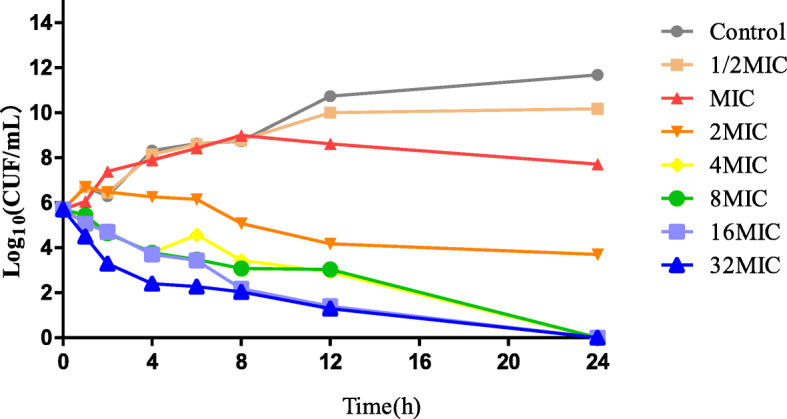
In vitro time-killing curves of ceftiofur against *Actinobacillus pleuropneumonia*

**Fig. 3:**
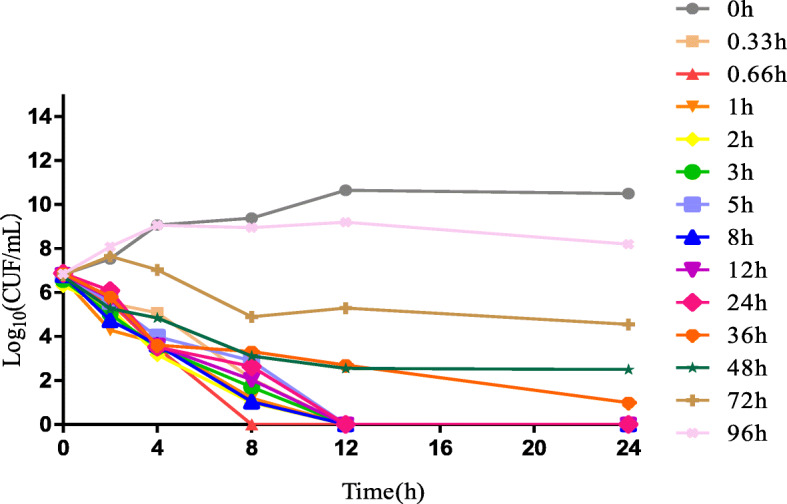
Ex vivo time-killing curves of ceftiofur against *Actinobacillus pleuropneumonia*

The time-killing curves of ceftiofur against *BW39* in vitro and ex vivo were shown in Figs. 2 and 3. When the drug concentration was 1/2 MIC-1 MIC, bacterial growth was not inhibited. Concentrations between 1 MIC and 2 MIC showed a slight inhibitory effect on the bacterial growth. Ceftiofur achieved a maximum bactericidal effect from 4 MIC-8 MIC. The ex vivo killing curve showed that the plasma collected between 0.33 and 2 h achieved the maximum bactericidal effect at the highest ceftiofur concentration. When the concentration was below the MIC at 96 h, ceftiofur no longer inhibited the growth of bacteria.

### Pharmacokinetic analysis of ceftiofur in plasma

**Fig. 4:**
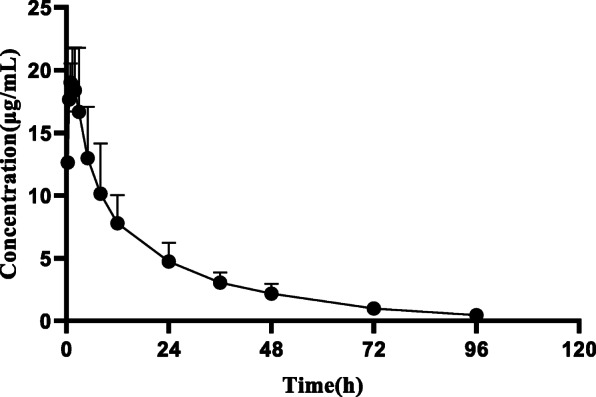
The concentration curve of DFC in healthy pigs after intramuscular injection 5 mg/kg of ceftiofur hydrochloride (*n* = 6)

LOD and LOQ of HPLC methods for detecting DFC were 0.03 μg/mL and 0.08 μg/mL, respectively. The linear range of the standard and working curve of DFC ranged from 0.1 to 50 μg/mL, with a coefficient of determination (R^2^) of 0.9999 and 0.9987. The recovery of ceftiofur in plasma ranged from 82.95 to 84.38%. The pharmacokinetic parameters calculated using non-compartment model analysis after I.M. (5 mg/kg BW) administration in healthy pigs are shown in Table 2 (Parameters of compartmental model is shown in Additional file 1: Table1). DFC concentrations were measured at different times shown in Fig. 4.

**Table 2:** Pharmacokinetic parameters of Ceftiofur after I. M administration (5 mg/kg) in healthy pigs

Parameters	Units	Healthy
AUC_0-24h_	μg·h/mL	358.84 ± 91.87
AUC_0-∞_	μg·h/mL	372.05 ± 97.35
C_max_	μg/mL	22.33 ± 3.17
T_max_	hr	0.66–2
T1/2	h	19.51 ± 2.76
Ke	h-1	0.04 ± 0.01
CL/F	mL/kg/h	282.49 ± 52.91
MRT_0-t_	h	22.82 ± 1.83
MRT_0-∞_	h	26.33 ± 2.94

### PK/PD integration and analysis

The PK/PD indices were determined by integrating the in vivo PK data and **the ex vivo time-killing curve**. The ratios of C max/MIC, AUC_24 h_/MIC, and T > MIC were 22.33 ± 1.98, 358.84 ± 57.42 h, and 72.2 ± 7.32 h, respectively. The values of the PK/PD index versus antibacterial effects in serum were simulated by the sigmoid E_max_ model. The model parameters of Hill coefficient, N, E max and AUC 24 h/MIC of the different levels are presented in Table 3. The values of the AUC24 h/MIC ratio required for bacteriostatic activity (E = 0), bactericidal activity (E = − 3), and bacterial elimination (E = − 4) were 45.73, 63.83, and 69.04 h.

**Table 3:** PK/PD analysis of data acquired from ex vivo time-killing curve of ceftiofur against a representative strain of *APP* BW39 in serums

Parameter	Unit	healthy
E_max_	Log_10_ CFU/ml	3.68
E_0_	Log_10_ CFU/ml	−6.88
E_max_-E_0_	Log_10_ CFU/ml	10.56
EC_50_	h	21.97
N	–	1.78
AUC 24 h /MIC for bacteriostatic	h	45.73
AUC 24 h /MIC for bactericidal	h	63.83
AUC 24 h /MIC for elimination	h	69.04

### Dose estimation

**Fig. 5:**
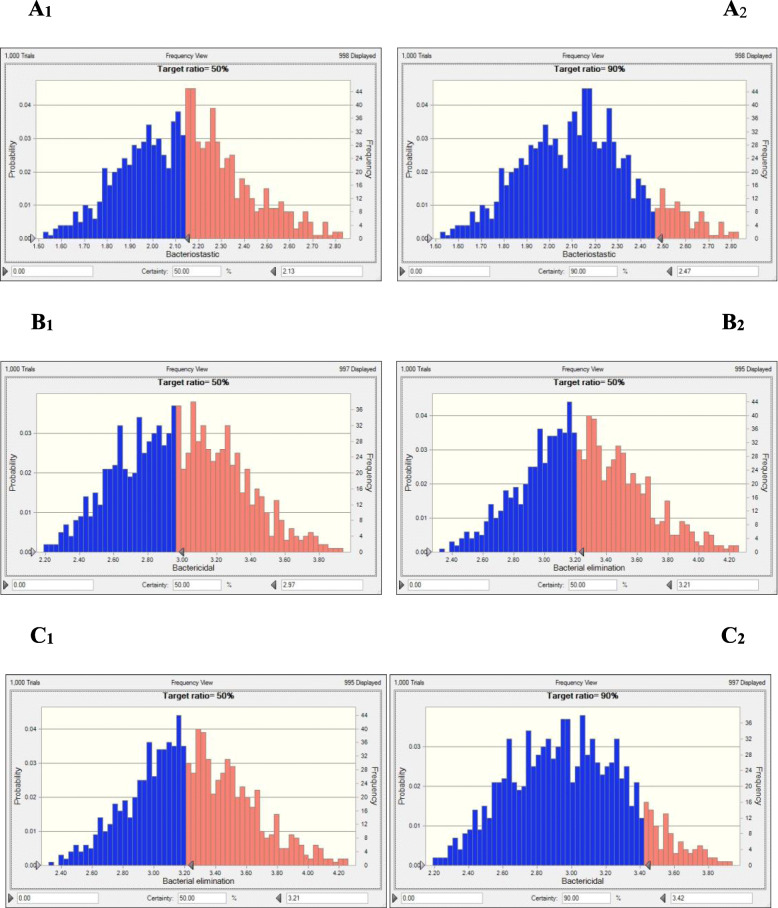
The predicted doses of ceftiofur curing A. *pleuropneumonia* for 50 and 90% TAR. A_1_:50% TAR of bacteriostatic effect; A_2_:90% TAR of bacteriostatic effect; B_1_:50% TAR of bactericidal effect; B_2_: 90% TAR of bactericidal effect; C_1_:50% TAR of elimination effect; C_2_: 90% TAR of elimination effect

According to the PK-PD integration model, the PK-PD index, AUC 24 h/MIC, was altered based on different outcomes in healthy swine. The prediction of ceftiofur curing *A. pleuropneumoniae* diseases for the 50 and 90% targets was calculated by the Monte Carlo simulation and dosage equation, which were shown in Fig. 5. The doses predicted to exhibit bacteriostatic, bactericidal, and elimination effects for *A. pleuropneumoniae* over 24 h were 2.13, 2.97, and 3.42 mg/kg for the 50% target attainment rate (TAR) and 2.47, 3.21, and 3.70 mg/kg for the 90% TAR, based on Crystal Ball software.

### CO_PD_ determination with Monte Carlo simulation

According to the MIC distribution, AUC_24 h_ derived from healthy pigs and the PK-PD target (AUC_24 h_/MIC)_ex_ of 63.83 h, 10,000 subjects were simulated by Monte Carlo simulation. The PTA (possibility target achievement) values were calculated as 88.94, 100 and 100% when the MICs (PK-PD cut- off) were defined as 4, 2 and 1 μg/ml, respectively (Table 4). Therefore, the CO_PD_ for ceftiofur against *Actinobacillus pleuropneumoniae* was defined as 2 μg/mL, for the current dose of 5 mg/kg by IM route.

**Table 4:** The AUC_24_/MIC values calculated with Monte Carlo simulation for PTA

Dose (mg/kg)	AUC (μg·h/mL)	MIC (μg/mL)			
		1	2		4
5	358.84 ± 91.87	100%	100%		88.94%

## Discussion

Ceftiofur, whether administered as hydrochloride or sodium salt, was metabolized rapidly to desfuroylceftiofur. The plasma half-life of ceftiofur sodium after intravenous dosing in swine was approximately 10 min [16]. The previous study [17] has tested the susceptibility of ceftiofur and its metabolite desfuroylceftiofur against *Actinobacillus pleuropneumoniae*(*n* = 50), the MIC90 of ceftiofur was 0.0078 μg/mL, and 0.015 μg/mL for desfuroylceftiofur, which suggested ceftiofur can be predictive of in vitro activity of ceftiofur and its major metabolite, desfuroylceftiofur. In our study, we determined the vitro susceptibility of ceftiofur, instead of desfuroylceftiofur, against 135 *Actinobacillus pleuropneumoniae* strains. The MIC values ranged from 0.0075 μg/mL to 4 μg/mL, and the MIC_50_ and MIC_90_ values were 0.015 μg/mL and 0.5 μg/mL, respectively, which were slightly different from the results of ceftiofur against 50 *Actinobacillus pleuropneumoniae* strains (MIC ranged from 0.0039–0.0015 μg/mL) [17].

The epidemiologic cutoff was established based on the pretest of drug susceptibility and then analyzed by statistical methods. At present, CLSI and EUCAST guidelines mainly involve analysis by nonlinear regression [18]. EUCAST based on the nonlinear regression complied the ECOFFinder software [19]. In this study, the epidemiologic cutoff was 0.125 μg/L according to the ECOFFinder results.

There are currently 18 serotypes of *Actinobacillus pleuropneumoniae* [2, 3]. The prevalent serotypes are different all over the world. The main prevalent serotypes in Canada are serotypes 1, 3 and 5, those in Europe are serotypes 1, 2, 5, 7 and 9 [20], and those in the United States are serotypes 1, 5 and 7 [21]. However, serotypes 1, 3,5 and 7 were the main prevalent serotypes in China [22]. Moreover, serotype 1 was more virulent than other serotypes [23, 24] Therefore, A strain *App* BW39, whose MIC was similar to MIC90(0.5 μg/mL) and serotype was serotype 1, was analyzed for PD study in broth and serum. According to the MIC and MBC values in vitro and ex vivo, the MBC/MIC ratio was 2 and 1. Therefore, ceftiofur is a bactericidal drug with an MBC/MIC ratio < 4 [25]. The results of in vitro and ex vivo killing curves showed, when the drug concentration was 1/2 MIC-1 MIC, bacterial growth was not inhibited. With the concentration increasing, the bactericidal effect increased and achieved the maximum bactericidal effect at 4MIC-8MIC. These findings suggest that ceftiofur has a time-dependent inhibition activity with a partly concentration-dependent pattern against *Actinobacillus pleuropneumoniae* both in vitro and ex vivo [26].

To establish a pharmacokinetic/pharmacodynamics cutoff, we needed to obtain the PK-PD parameters, MIC distribution and pharmacodynamic targets. The most appropriate PK/PD index to determine the antibacterial efficacy and predict the therapeutic efficacy for β-lactam antibiotics is ƒ%T > MIC [9]. The killing curve showed that ceftiofur had a time-dependent inhibition activity same with a partly concentration-dependent pattern against *Actinobacillus pleuropneumoniae*. Moreover, PK/PD index depends on the shape of the plasma exposure curve which may differ widely between the many modalities of Antimicrobial drug administration, such as antimicrobial drug was incorporated in food or drinking water or administrated by oral. VetCAST suggested using AUC/MIC as a default index. The index of the AUC/MIC ratio was also used to describe the character of the antibacterial activity of time-dependent killing with prolonged persistent efficacy [27]. In our manuscript, the PK/PD index (AUC/MIC), using an inhibitory sigmoidal Emax model, showed a favorable correlation (r^2^ = 0.9967) with the predicted antibacterial efficacy. For these reasons, the AUC/MIC parameter was regarded as the most appropriate PK/PD index to describe the antibacterial activity of ceftiofur in serum. An AUC/MIC ratio > 125 h is generally considered the best activity indicator [28, 29]. However, the target parameters might be different for various drugs, target organisms or bacteria [30]. For example, the AUC_24 h_/MIC values for three levels of preventive, therapeutic, and bacterial eradication were 28.40, 29.51 and 29.13 h, respectively, in previous study on PK/PD modeling of Ceftiofur Sodium against *Haemophilus parasuis* infection in pigs [26]. In another study, the AUC_24 h_/MIC values for three levels of preventive, therapeutic, and bacterial eradication were 24.6, 43.8 and 58.4 h, respectively [31]. Prior to our study, no PK-PD integration modeling analyses were performed for ceftiofur against *Actinobacillus pleuropneumoniae*. In our study, the ex vivo values of AUC_24 h_/MIC of ceftiofur against bacteriostatic, bactericidal and eradication were 45.73, 63.83 and 69.04 h. Through Monte Carlo simulations, when the MIC values were 2 μg/mL and 4 μg/mL, the probability of target attainment was 100 and 88.94%, respectively. Therefore, the CO_PD_ was calculated as 2 μg/mL, for the current dose of 5 mg/kg by IM route.

Base on non-compartmental model, the peak concentration reached 22.33 ± 3.17 μg/mL at 0.66–2 h, the elimination half-time was 19.51 ± 2.76 h, and AUC was 358.84 ± 91.87 μg·h/mL, which was similar to previous study [3]. The PK of ceftiofur may differ from that of diseased pigs. Previous study has demonstrated that infected pigs may have higher values of Vz/F and CL/F, but lower values of AUC, MRT and t1/2z compared with non-infected pigs.

PD-PD integration modeling can be used to select rational dose regimes in veterinary medicine [29]. Based on the dose estimation equation and Monte Carlo simulation, the ‘fu’ in the equation was the free fraction of the drug in plasma. Ceftiofur had a high protein binding rate, which was 90% in cow and adult cattle [32, 33],The EMA [34] reported that the protein binding of ceftiofur and its metabolite DFC in swine was 0.7, which was applied in this research. According to the MIC distribution in China, the predicted daily doses for the 50 and 90% targets of ceftiofur with bactericidal activity against *Actinobacillus pleuropneumoniae* were 2.97 and 3.21 mg/kg body weight, respectively.

Generally, Clinical breakpoint is determined by epidemiological cut-off value, PK/PD breakpoint and Clinical cutoff [24, 25]. Under the clinically recommended dose (5 mg/kg), the ceftiofur CO_PD_ value (2 μg/mL) against *A. pleuropneumonia* was higher than the ECOFF value (0.125 μg/mL). It probably means that the current dose (5.0 mg/ kg) could guarantee clinical efficacy to treat the wild-type populations of *A. pleuropneumoniae*. In fact, our calculated accurate dose of ceftiofur hydrochloride for a PTA ≥ 90% was 3.21 mg/kg. Previous study [3] has shown the similarity of the pharmacokinetic parameters of the sodium and hydrochloride formulations of ceftiofur, similar therapeutic efficacy therefore can be inferred for the two products. So, considering the MIC distribution in China, the dose of ceftiofur could be 3.21 mg/kg, However, it is practically difficult to determine a clinical cutoff in veterinary medicine [24, 26]. Owing to the paucity of relevant data to bridge the relationship between MIC and clinical cure. In our study, CO_PD_ values contains the ECOFF values, and the CO_PD_ (2 μg/mL) will therefore be recommended as surrogate.

## Conclusions

In conclusion, we reveal the EOFF and PK/PD cutoff values of ceftiofur against A. pleuropneumoniae in piglets. However, with the paucity of clinical data for ceftiofur to establish a clinical cutoff against A. pleuropneumoniae, the PK/PD cutoff value of 2 μg/mL will be recommended as surrogate. Moreover, according to the PK/PD data and the MIC distribution in China, the single bactericidal dose was 3.21 mg/kg for the 90% target, which would be more able to cure *Actinobacillus pleuropneumoniae* and avoid the emergence of resistance for clinical ceftiofur use of ceftiofur in piglet.

## Methods

### Chemicals and reagents

The standard ceftiofur hydrochloride (det. Purity: 97.9%) purchased from Dr. Ehrenstorfer (Augsburg, Germany) and 5% ceftiofur hydrochloride injection purchased from Pfizer Animal Health Co., Ltd., Canada, were used during the study. All the chemical reagents used were HPLC grade.

### Bacterial strain isolation

*E. coli* ATCC 25922 was purchased from American Type Cell Culture and used as a quality control strain for drug susceptibility testing. *Actinobacillus pleuropneumoniae* serotype 1 (BW39) was used to determine the EOFF and CO_PD_ values. A total of 101 *Actinobacillus pleuropneumoniae* strains were donated by the International Research Center for Animal Diseases, China State Key Laboratory of Agricultural Microbiology, College of Veterinary Medicine, Huazhong Agricultural University. Thirty-four *Actinobacillus pleuropneumoniae* strains were isolated from the tissue of infected pigs. The strains were stored at − 80 °C prior to each experiment. Prior to testing, each isolate was subcultured at least three times in TSA and TSB containing 5% newborn calf serum and 1% NAD (Zhejiang Tianhang Biotechnology Co., Ltd.).

### Determination of antimicrobial susceptibility and epidemiologic cutoff

The susceptibility of 135 *Actinobacillus pleuropneumoniae* strains to ceftiofur was determined by inoculating the strains on TSA agar plates supplemented with newborn calf serum and 1% NAD and incubating the plates at 37 °C in an atmosphere containing 5% CO_2_ for 24 h; the susceptibility was measured using the standard agar dilution method with concentrations of ceftiofur between 0.00375–32 μg/ml, according to CLSI [35] protocols. The MIC distribution was constructed and converted into a cumulative log-normal distribution. Then, nonlinear regression was employed to fit the cumulative log_2_-transformed MIC data to obtain a range of optimum wild-type MIC distributions, which contained the wild-type MIC in the range of 0.1 and 99.9%, and to calculate the probability of MIC data falling within the cutoff range. The optimum fit was defined as the fit where the difference between the estimate of the isolate number and the actual number was minimal. The cutoff value would encompass at least 95% of the wild-type isolates [18]. A wild-type cutoff (CO_WT_) was developed based on the MIC distribution following CLSI M37-A3. The CO_WT_ value was calculated by ECOFFinder software (J. Turnidge, Kahlmeter, and Kronvall 2006), which is available on the CLSI website (https://clsi.org/education/microbiology/ecoffinder/).

### Pharmacodynamics of ceftiofur against *BW39*

#### MIC, MBC and MPC determination

The minimum inhibitory concentration (MIC) of ceftiofur against BW39 (serotype 1) was determined by the broth micro dilution method according to the CLSI (Clinical and Laboratory Standards Institute, 2015) and in serum, as vitro MIC and ex vivo MIC. The MIC was determined as the lowest ceftiofur concentration that visibly inhibited the growth of bacteria at the end of the 24 h incubation period. For the MBC of ceftiofur against BW39, 100 μL from each well was subjected to 10-fold or more dilution with broth and serum, as vitro MBC and ex vivo MBC; 10 μL of each solution was spread on TSA agar plates and incubated at 37 °C for 24 h for colony forming unit (CFU) counting. The MBC was defined as the lowest drug concentration that resulted in a 99.9% reduction in the bacterial density. The mean was expressed as the final result.

The mutant prevention concentration (MPC) was determined by the agar method according to the procedures of Blondeau [36]. Exponential growth phase bacteria were pelleted by centrifuging at 3000 rpm at 4 °C. The pellet was then diluted to 3 × 10^10^ CFU/mL with TSB medium. An aliquot of 100 μL of the 10^10^ CFU/mL bacterial suspension was cultured on TSA agar plates containing various concentrations of ceftiofur (0 × MIC, 1 × MIC, 2 × MIC, 4 × MIC, 8 × MIC, 16 × MIC, 32 × MIC, and 64 × MIC) obtained from a series of two-fold dilutions. Inoculated plates were incubated for 72 h, and colonies were counted every 24 h. All MPC determinations were performed in duplicate. The MPC was defined as the lowest ceftiofur concentration with no visible bacterial growth on agar plates after incubation for 72 h.

#### Bacterial growth and time-killing curve of BW39 in vitro and ex vivo

The BW39 isolate was selected to determine the growth curve and time-killing curve in TSB broth and serum. The in vitro and ex vivo growth curves of the BW39 isolate were established by plotting time versus log_10_ CFU/mL. An aliquot of 5 mL of BW39 grown to mid-log phase with a starting inoculum of 10^6^ CFU/mL was added to 5 mL of TSB broth supplemented with serial concentrations of ceftiofur corresponding to 0 × MIC, 1 × MIC, 2 × MIC, 4 × MIC, 8 × MIC, 16 × MIC, and 32 × MIC for the in vitro time-killing curve. In addition, an aliquot of 5 mL of BW39 grown to the mid-log phase with a starting inoculum of 10^6^ CFU/mL was co-incubated with 5 mL sterilized blank serum added with ceftiofur corresponding to the concentration of ceftiofur in serum,which was collected from healthy pigs at different time points (0, 0.33, 0.66, 1, 1.5, 2, 3, 5, 8, 12, 24, 36, 48, 72 and 96 h) after I.M. administration of a single injection of 5 mg/kg ceftiofur hydrochloride for the determination of the ex vivo time-killing curve. The tubes were incubated at 37 °C with 5% CO_2_. Each culture was serially diluted 10-fold with sterile saline, and 100 μL of each dilution was spread onto TSA agar plates at different time points (0, 2, 4, 6, 8, 12, and 24 h). Then, the bacterial count (CFU/mL) was determined after incubation for 24 h at 37 °C with 5% CO_2_. The limit of detection was 10 CFU/mL. The in vitro and ex vivo time-killing curves of ceftiofur against BW39 were established by plotting the time versus log_10_ CFU/mL. The experiment was tested in triplicate.

#### In vitro PAE determination

Approximately 1.8 mL exponential phase *A.pleuropneumoniae* BW39 (1.0 × 10^7^ CFU/mL) was mixed with 0.2 mL ceftiofur, to generate final concentrations of 1 MIC, 2 MIC and 4 MIC. A 0.2 mL aliquot of physiological saline was used as control. Volumes were cultured in glass tubes and grown for 1 h and 2 h to induce PAE production. One hundred microliter cultured medium was mixed with 0.9 mL TSB medium and cultured 24 h at 37 °C with 5% CO_2_. One hundred microliter samples were taken at 0, 1, 2, 4, 6, 8, and 12 h, and serially diluted 10-fold with sterile physiological saline to count cells. Each treatment was performed four times. Growth curve for *A.pleuropneumoniae* BW39, at different ceftiofur concentrations were established, and T (time required for bacterial numbers to be 10 times higher than 0 h in the test groups) and C (time required for the bacterial numbers to be 10 times higher than 0 h in control groups) values were calculated. PAE was calculated as the difference between T and C (PAE = T - C) [26].

### Pharmacokinetics of ceftiofur in plasma of pigs

#### Animals

Six 6-week-old healthy castrated crossbred piglets (Duroc×Landrace×Yorkshire) pigs with an average weight of 15 ± 2 kg were purchased from the Livestock and Poultry Breeding Center of Hubei Province (Wuhan, China). The animals were acclimatized for a period of 1 week before the experiment. The temperature and relative humidity of the housing environment were kept at 18–25 °C and 45–65%, respectively. All animal experiment procedures were approved by the Institutional Animal Care and Use Committee at Huazhong Agricultural University (HZAUSW-2016-007).

#### Samples collection

Six pigs were received ceftiofur hydrochloride injection at a dose of 5 mg/kg·b.w by intramuscular injection of neck. Blood samples (5 mL) from each pig of each group were gently collected from the jugular vein at 0, 0.33, 0.66, 1, 1.5, 2, 3, 5, 8, 12, 24, 36, 48, 72 and 96 h. Plasma samples were obtained by centrifuging the blood at 3500 rpm/min for 10 min, and the samples were stored at − 20 °C prior to the analysis and analysis within 3 days after sampling.

#### Sample analysis

DFC concentration were measured by HPLC described by previous study [26] to represent ceftiofur plasma concentration, as ceftiofur is rapidly metabolized to DFC in piglets.

Extraction: 0.5 mL plasma was mixed with 7 mL of 0.4% DTE-borate buffer. The mixture was incubated for 15 min at 50 °C in a water bath, with a 10 s vortexing every 3 min. Samples were then centrifuged after cooling to 25 °C, then the supernatant was collected.

Solid phase extraction: An Agilent HLB column (60 mg/3 cc) was activated and equilibrated consecutively with 3 mL methanol and ultrapure water. Extracted materials were added to the HLB column and a flow rate set at 1 mL/min. The column was then eluted with 5 mL methanol, after which the eluate was concentrated by nitrogen at 35 °C. The concentrated solution was vortexed with 0.5 mL ultrapure water, sonicated for 5 min. The DFC standard was added to 0.5 mL plasma (to achieve final concentrations of 0.1, 0.25, 0.5,1.0, 2.5, 5.0, 10.0, 20.0 and 50 μg/mL) and prepared with same process as samples from test groups.

The quantitative analysis of ceftiofur in plasma was performed with a Water 2695 series HPLC with a UV detector at a wavelength of 266 nm. A ZORBAX Stable Bond-C18 column (250 mm × 4.6 mm, i.d. 5 μm, Agilent) was used to achieve chromatographic separation. The mobile phase consisted of 0.1% trifluoroacetic acid (phase A) and acetonitrile (phase B) at a flow rate of 1 mL/min at 30 °C with isometric elution conditions (86,14, v/v).

The concentration-time data for ceftiofur in plasma samples harvested from healthy pigs were analyzed by WinNonlin 5.2.1 software (Pharsight Corporation, Mountain View, CA, USA) to obtain the pharmacokinetic parameters.

#### Pharmacokinetic/pharmacodynamic integration and dose estimations

There are three standard indices (ƒ%T > MIC, ƒC_max_/MIC, and ƒAUC_0–24 h_/MIC) for an antibiotic [37]. The inhibitory sigmoid Emax model (Hill equation) was analyzed the integration of AUC24h/MIC ratio in vitro and bacteria count change (CFU/ml) in serum during 24 h incubation. The model equation was described as follows:

$$ E={E}_{\mathrm{max}}-\frac{\left({E}_{\mathrm{max}}-{E}_0\right)\cdot {C}^N}{C^N+{EC}_{50}^N} $$

where *E* is the antibacterial effect measured as the change in the bacterial count (log_10_ CFU/mL) in plasma sample after 24 h of incubation compared with the initial incubation, *E*_*0*_ is the change in log_10_ difference in bacterial count in the control sample after 24 h of incubation; *E*_*max*_ is the maximum antibacterial effect determined as the difference in log_10_ CFU/mL in the sample after the incubation, *EC*_*50*_ is the PK/PD parameter value producing 50% of the maximum antibacterial effect; *C* is the PK/PD parameter value in the effect compartment (the ex vivo site, that is plasma); and *N* is the Hill coefficient, which describes the steepness of the PK/PD parameter-effect curve.

PK/PD parameter values corresponding to the E value (derived from the sigmoid E_max_ equation) in plasma were used to deduce an optimal dose regimen. The potential optimal dosage was calculated using the following equation:

$$ Dose=\frac{{\left({AUC}_{24 th}/ MIC\right)}_{ex}\times MIC}{fu}\times CL/F $$

where *MIC* is the ex vivo minimum inhibitory concentration; (AUC_24 h_/MIC)_*ex*_ is the target end point for optimal efficacy; *CL* is the clearance; *fu* is the free fraction of ceftiofur in plasma; and F is the bioavailability of ceftiofur.

The distribution probabilities for predicted daily dosage were performed to achieve simulated 50 and 90% TAR under 1000 trials with Crystal Ball software (*version 11.1.2*, Oracle, United States).

#### Monte Carlo simulation and the pharmacokinetic/pharmacodynamics cutoff (CO_PD_)

A Monte Carlo simulation (MCS) with 10,000 iterations was conducted using Crystal Ball software (version 7.2.2) (Oracle, United States) based on PK parameters and calculated PK/PD targets (AUC_24h_/MIC) when each possible MIC and the target AUC_24h_/MIC achieving a bactericidal action (E = − 3). The AUC_24h_ was assumed to be normally. CO_PD_ was defined as the maximal MIC value at which the corresponding PTA was ≥90% [38].

## Supplementary information

**Additional file 1 **Table 1: Compartmental pharmacokinetic parameters of ceftiofur in plasma by 5 mg/kg IM (*n* = 6)

## Data Availability

The datasets used and/or analyzed in this study are available from the corresponding author on reasonable request.

## Abbreviations

APP: *Actinobacillus pleuropneumoniae*
AST: Antimicrobial susceptibility testing
AUC/MIC: The area under the concentration-time curve to MIC ratio
CLSI: Clinical & Laboratory Standards Institute
CO_PD_: PK/PD (pharmacokinetic/pharmacodynamic) cutoff value (named by EUCAST as the PK/PD breakpoint)
DFC: Desfuroylceftiofur
EMA: European Medicines Agency
ECOFF: Epidemiological cutoff value [synonym of wild-type cutoff value (COWT)]
EUCAST: European Committee on AST
HPLC: High performance liquid
LOD: Limited of Detection
LOQ: Limited of Quantitation
MBC: Minimal bactericidal concentration
MIC: Minimal inhibitory concentration
NAD: Nicotinamide Adenine Dinucleotide PAE Post-antibiotic effect
PK/PD: Pharmacokinetic/pharmacodynamic
PTA: Probability of target attainment
T > MIC: The cumulative percentage of a 24 h period that the drug concentration exceeds the MIC under steady-state pharmacokinetic conditions unless otherwise stated
TSA: Tryptic Soy Agar
TSB: Tryptic Soy Broth
