# Supplementary material for: Optimal regimens based on PK/PD cutoff evaluation of ceftiofur against Actinobacillus pleuropneumoniae in swine
Source: BMC Vet Res. 2020 Sep 29;16:366. doi: 10.1186/s12917-020-02589-9 (PMC7526406; doi:10.1186/s12917-020-02589-9)
Supplement: Supplementary file 1 — Additional file 1 Table 1: Compartmental pharmacokinetic parameters of ceftiofur in plasma by 5 mg/kg IM (n = 6) [file 12917_2020_2589_MOESM1_ESM.docx]

**Table1 Compartmental pharmacokinetic parameters of ceftiofur in plasma by 5 mg/kg IM (n=6)**

| Parameters | Units | Healthy |
| --- | --- | --- |
| AUC | μg·h/mL | 364.34±105.91 |
| Cmax | μg/mL | 20.3±2.75 |
| Tmax | hr | 0.66-2 |
| α | 1/h | 0.5±0.35 |
| β | 1/h | 0.03±0.01 |
| T_1/2α_ | h | 2.74±2.81 |
| T_1/2β_ | h | 21.76±5.59 |
| K01 | h | 1.92±0.7 |
| K10 | h | 0.09±0.03 |
| K12 | h | 0.25±0.22 |
| K21 | h | 0.20±0.12 |
| CL/F | L/kg/h | 0.015±0 |
| V_Z_/F | L/kg | 0.20±0.08 |
